# MicroRNA-125b as a valuable predictive marker for outcome after autologous hematopoietic stem cell transplantation

**DOI:** 10.1186/s12885-023-10665-0

**Published:** 2023-03-03

**Authors:** Mohammad Rafiee, Fatemeh Amiri, Mohammad Hossein Mohammadi, Abbas Hajifathali

**Affiliations:** 1 Department of Medical Laboratory Sciences, School of Paramedical Sciences, Zanjan University of Medical Sciences, Zanjan, Iran; 2 Department of Medical Laboratory Sciences, School of Paramedicine, Hamadan University of Medical Sciences, Hamadan, Iran; 3 Clinical Research Development Unit of Shahid Beheshti Hospital, Hamadan University of Medical Sciences, Hamadan, Iran; 4 Department of Hematology and Blood Banking, School of Allied Medical Sciences, Shahid Beheshti University of Medical Sciences, Tehran, Iran; 5 Hematopoietic Stem Cell Research Center, Shahid Beheshti University of Medical Sciences, Tehran, Iran

**Keywords:** Autologous hematopoietic stem cell transplantation, Relapse, Lymphoma, Multiple myeloma, miR-125b

## Abstract

**Background:**

Relapse is a frequent occurrence in autologous hematopoietic stem cell transplantation (AHSCT), and early relapse after AHSCT results in poor survival and low quality of life. Predictive marker determination for AHSCT outcomes could be helpful in the prevention of relapse through personalized medicine. Here the predictive value of circulatory microRNAs (miRs) expression for AHSCT outcomes was studied.

**Methods:**

50 MM and lymphoma candidates for AHSCT participated in this study. Two plasma samples were obtained before AHSCT from each candidate; one before mobilization and the other after conditioning. Extracellular vesicles (EVs) were isolated by ultracentrifugation. miR-125b, miR-126, miR-150, and miR-155 expression were analyzed in both plasma and EVs using real time polymerase chain reaction analysis. Other data related to AHSCT and its outcomes were also collected. The predictive value of miRs and other factors for outcomes was assessed by multi-variant analysis.

**Results:**

By 90 weeks follow up after AHSCT, multi-variant and ROC analysis showed miR-125b as a predictive marker for relapse, high lactate dehydrogenase (LDH), and high erythrocyte sedimentation rate (ESR). The cumulative incidence of relapse, high LDH, and high ESR increased with an increase in circulatory miR-125b expression.

**Conclusion:**

miR-125b could be applicable in prognosis evaluation and also create a possible new targeted therapy opportunity for enhanced outcomes and survival after AHSCT.

**Trial registration:**

The study was retrospectively registered. Ethic code No: IR.UMSHA.REC.1400.541.

## Background

Many years have passed since autologous hematopoietic stem cell transplantation (AHSCT) or rescue therapy was considered the primary and effective therapeutic approach to multiple myeloma (MM), lymphoma, and other neoplasms [1, 2]. Relapse is one of the leading causes of treatment failure after stem cell transplantation [3]. While some patients get long-term remissions and rarely, even cure, the prognosis for high-risk MM patients remains poor. Treatment response and early relapse after AHSCT can be affected by characteristics associated with patients or diseases, such as weakness, clinical stage, age, cytogenetic abnormalities, and comorbidities [4]. Early relapse will decrease survival in standard and high-risk MM patients [4]. The relapsed cases have so heterogeneous pattern of clinical expression. Therefore, a personalized approach should be taken for treatment in transplantation time [5]. Also, recurrence of Hodgkin lymphoma after AHSCT occurs in 50% of cases in a year [6]. Relapse after AHSCT in non-Hodgkin lymphoma like follicular lymphoma is common and treatment line, age, graft purging, and myeloablative regimens are considered effective factors in it [7].

MicroRNAs (miRNAs or miRs) as small non-coding RNAs have critical and wide roles in the expression of genes and also cellular function including cell cycle, growth, differentiation, apoptosis, and so on, either in normal or in the malignant form [8]. Many of miRNAs have been used as biomarkers for early detection, target therapy, and follow-up in various diseases like lymphoma [9]. For instance, up-regulation of miR-125b results in down-regulation of its target i.e., SRY-Box Transcription Factor 30 (SOX30) in malignant lymphomas that can be regarded as a useful biomarker, diagnostically and therapeutically [10]. Poor clinical outcomes are associated with elevated expression of mir-126 in angioimmunoblastic T-cell lymphoma [11]. miR-150 increases the sensitivity of natural killer/ T (NK/T) cell lymphoma to ionizing radiation through suppression of phosphoinositide 3-kinases/ serine-threonine protein kinas/ mechanistic target of rapamycin kinase (PI3K/AKT/mTOR) pathway [12] and in MM it has seen that less expression of miR-155 related to resistance to bortezomib [13]. miRNAs can be packaged in membranous enclosed structures, known as extracellular vesicles (EVs), carried to definite targets via plasma, and protected from environmental factors [14].

Here, we were going to find a predictive marker for outcomes after AHSCT like relapse or signs of relapse before being evident. In our center, many variables related to patients and microRNA expression are considered in this regard. Given to importance of miRNAs, especially those in EVs, in this study the predictive value of miR-125b, miR-126, miR-150, and miR-155 expression in plasma and EVs, along with many outcome-related data from AHSCT candidates has been evaluated.

## Methods

### Patients and sample collection

50 MM and lymphoma candidates for AHSCT in the department of bone marrow transplantation center, Taleghani hospital (Tehran, Iran) were selected between September 2021 and August 2022. All protocols and blood sampling were conducted after filling out the informed consent form by patients and confirmation in ethics committee of Hamadan university of medical sciences (Ethic code No: IR.UMSHA.REC.1400.541). Mobilization was done by granulocyte colony-stimulating factor (G-CSF) administration in a steady state. After mobilization and leukocyte count reaches to the appropriate number, for HSCs collection from peripheral blood, Spectra Optia Apheresis System was applied. Lomustine, Etoposide, Cytarabine, Melphalan (CEAM) or Carmustine, Etoposide, Cytarabine, Melphalan (BEAM), and Melphalan/Velcade were conditioning regimens for lymphoma and MM patients, respectively. Patients’ available data was collected from diagnosis to transplantation phase and almost 90 weeks (as the median) follow-up from medical records and new laboratory tests that were done on the patients. 2–4 milliliters of peripheral blood samples were obtained in EDTA containing tube at two times. The first sample was 1–2 days before mobilization and the second sample was 1–2 days after conditioning and before HSCs re-infusion (post conditioning sample).

### EVs isolation and confirmation

Based on our previous study [15], EV isolation was performed by ultracentrifugation. Plasma was isolated by centrifugation at 1000 g for 10 min. 1/1 dilution of plasma with phosphate buffer solution (PBS) was prepared and then centrifugation was performed for 10 min at 2000 g to precipitate dead cells. Subsequently, the supernatant was re-centrifuged for 30 min at 10,000 g to discard cell debris. The obtained supernatant was ultracentrifuged at 100,000 g for 80 min and after the discard of the supernatant, precipitated EVs were diluted by filtered PBS. Then precipitated EVs were filtered through a 0.2 μm filter to remove apoptotic bodies. Finally, ultracentrifugation was done again like before on it. For confirmation of isolated EVs, CD9 and CD63 markers were evaluated by flow cytometry (Dako antibodies, Denmark, and Attune™ NXT Flow Cytometer). Their size was measured by dynamic light scatter (DLS) (Zeta-sizer, Malvern, UK) and the transmission electron microscopy (TEM) technique was done for morphology visualization of them.

### Circulatory microRNAs extraction and expression analysis

Expression of miR-125b, miR-126, miR-150, and miR155 in plasma and EVs, before mobilization and post conditioning were analyzed by real time polymerase chain reaction (PCR). The miR extraction was done on MVs/plasma by GeneAll RiboEx™ LS Kit (GeneAll Biotech, south Korea) based on the manufacturer’s instruction. cDNA was synthesized using ExcelRT ™ Reverse Transcriptase kit (SMOBio, South Korea) according to the manufacturer’s instruction and stem-loop primer (Metabion, Germany). In the expression analysis step, real time PCR reaction was performed in the total volume of 15 µl including 7.5 µl of Master mix 2× (Real Q Plus Master Mix, Denmark), 0.5 µl of each primer, 3 µl of the synthesized cDNA, and 3.5 µl of nuclease-free water in Corbett Rotor-Gene 600 thermocycler (Germany). The reactions were done in this condition: initial 95 °C for 15 min, 40 cycles of denaturation at 95 °C for 10s, annealing at 53–54 °C for 15s, extension at 72 °C for the 20s, and 10 min for the melting curve. miR-16 and SNORD47 geometric mean cycle threshold(CT)was used to calculate basal expression or 2^−ΔCT^. Sequences of a housekeeping gene, stem-loop, and miRNAs primers were shown in Table 1.

**Table 1:** Primers for expression of microRNAs and stem loop for cDNA synthesis

miRs	Sequences
**miR-125b (5P)**	5-GATGTCCCTGAGACCCTAA-3
**miR-126 (3P)**	5-GACAGTCGTACCGTGAGTA-3
**miR-150 (5P)**	5-GTATGTCTCCCAACCCTTG-3
**miR-155 (5P)**	5-GCTCAGTTAATGCTAATCGTG-3
**miR-16 (5P)**	5-GACAGTAGCAGCACGTAAAT-3
**SNORD47**	5-CCAATGATGTAATGATTCTGCC-3
**Reverse**	5-GAGGAAGAAGACGGAAGAAT-3
**Stem loop**	5GAAAGAAGGCGAGGAGCAGATCGAGGAAGAAGACGGAAGAATGTGCGTCTCGCCTTCTTTCHVMHNN-3

### Patients follow up

After 16–130 weeks, almost 90 weeks (as the median), all of the patients were followed about their outcomes. Factors including complete blood count (CBC) parameters, erythrocyte sedimentation rate (ESR), C-reactive protein (CRP), urea, creatinine, lactate dehydrogenase (LDH), calcium, alkaline phosphatase, total protein, gamma globin, M protein spike, β2 macroglobulin, lytic lesion in bone, plasma cell percent in bone marrow, and clinical features like lymphadenopathy, bone pain, weakness, headache, and fever as outcomes were evaluated and recorded from medical reports and new laboratory tests that were done on the patients. These factors are related to remission and relapse.

### Statistical analysis

All of the collected data from diagnosis to transplantation and follow-up time were analyzed in Poisson multivariate analysis to find predictive markers for all outcome factors using South Texas Art Therapy Association STATA software (College Station, Texas, USA, V14). Significant hazard ratio (HR) and p value show how often relapse happen over time in a dysregulated parameter. Evaluation of the predictive value for variables in the prediction of outcomes was analyzed by receiver-operating characteristic (ROC) analysis. In addition, Kaplan-Meier was used to estimate the cumulative incidence (CI) of outcomes and relapse free survival (RFS) analysis by effective variables. p value less than 0.05 was considered statistically significant.

## Results

### Patients’ data

50 candidates of AHSCT, with a mean and standard deviation of age 42.4 ± 14.6 years and 50% of them were female, were incorporated into the study. The median time for the engraftment of neutrophils and platelet was 11 days after AHSCT. Demographic data, CD34^+^ cell count/kg harvested by mobilization and apheresis, mobilization day number, the total dose of G-CSF, neutrophil and platelet count at apheresis day, and other AHSCT-related data were recorded (Table 2). The median follow-up was 90 weeks (range 16–130 weeks) and nine patients relapsed. In Table 2, other recorded variables as outcomes were shown.

**Table 2:** Participants criteria (N = 50)

Variables at the AHSCT time		Outcome variables	
Features	Median (Rang) / Frequency (%)	Features	Median (Rang) / Frequency (%)
**Age (year)**	40.5 (18–70)	**Hemoglobin (g/dl)**	12.3 (8.8–16.2)
**Gender**		**WBC (×10** ^**3**^ **/µl)**	5.03 (2.8–10.2)
Male	25 (50%)	**PLT (×10** ^**3**^ **/µl)**	178 (133–295)
Female	25 (50%)	**ESR (mm/hours)**	13.5 (2–82)
**Diagnosis**		**LDH (U/L)**	287 (148–450)
Multiple myeloma	24 (48%)	**Ca (mg/dl)**	9.3 (7.8–13)
Hodgkin lymphoma	15 (30%)	**Cr (mg/dl)**	1.03 (0.6-2.0)
Non-Hodgkin lymphoma	11(22%)	**ALP (IU/L)**	102 (51–373)
**BMI after transplantation**	27.1 (14.8–42.1)	**Urea (mg/dl)**	18 (11–23)
		**Relapse**	9 (18%)
**Mobilization day number**	6 (5–9)	**Positive lytic lesion** **(In MM patients)**	2 (8%)
**Total dose of infused G-CSF for mobilization (µg)**	3750 (1500–7200)	**Positive M protein spike**	4 (16%)
**WBC count at start day of mobilization (×10** ^**3**^ **/µl)**	6.05 (2.4–22.1)	**Positive radiologic signs in lymphoma patients**	6 (23%)
**Last injection of G-CSF to apheresis (hour)**	6 (1–22)		
**Neutrophil count on apheresis day (×10** ^**3**^ **/µl)**	35.13 (10.7–94.5)		
**Apheresis duration (min)**	377.5 (270–520)		
**Total processed blood in apheresis (Littr)**	17.97 (10.4–25.5)		
**WBC count in apheresis product (×10** ^**8**^ **/kg)**	10.9 (4.2–26.2)		
**MNC count in apheresis product (×10** ^**8**^ **/kg)**	6.20 (3.3–17.5)		
**CD34** ^**+**^ **cell count/kg (×10** ^**6**^ **/kg)**	3.5 (1-15.4)		
**CD34** ^**+**^ **in PAPBS (/µl)**	35 (4.1–116)		
**Platelet engraftment day**	11 (9–19)		
**Platelet count at engraftment day (×10** ^**3**^ **/µl)**	28 (20–110)		

**BMI**: Body mass index, **WBC**: White blood cell, **MNC**: Mono nuclear cell, **PAPBS**: Pre-apheresis peripheral blood sample, **ESR**: Erythrocyte sedimentation rate, **LDH**: Lactate dehydrogenase, **Ca**: Calcium, **Cr**: Creatinine, **ALP**: Alkaline phosphatase

### Isolated EV’s characterization

**Fig. 1:**
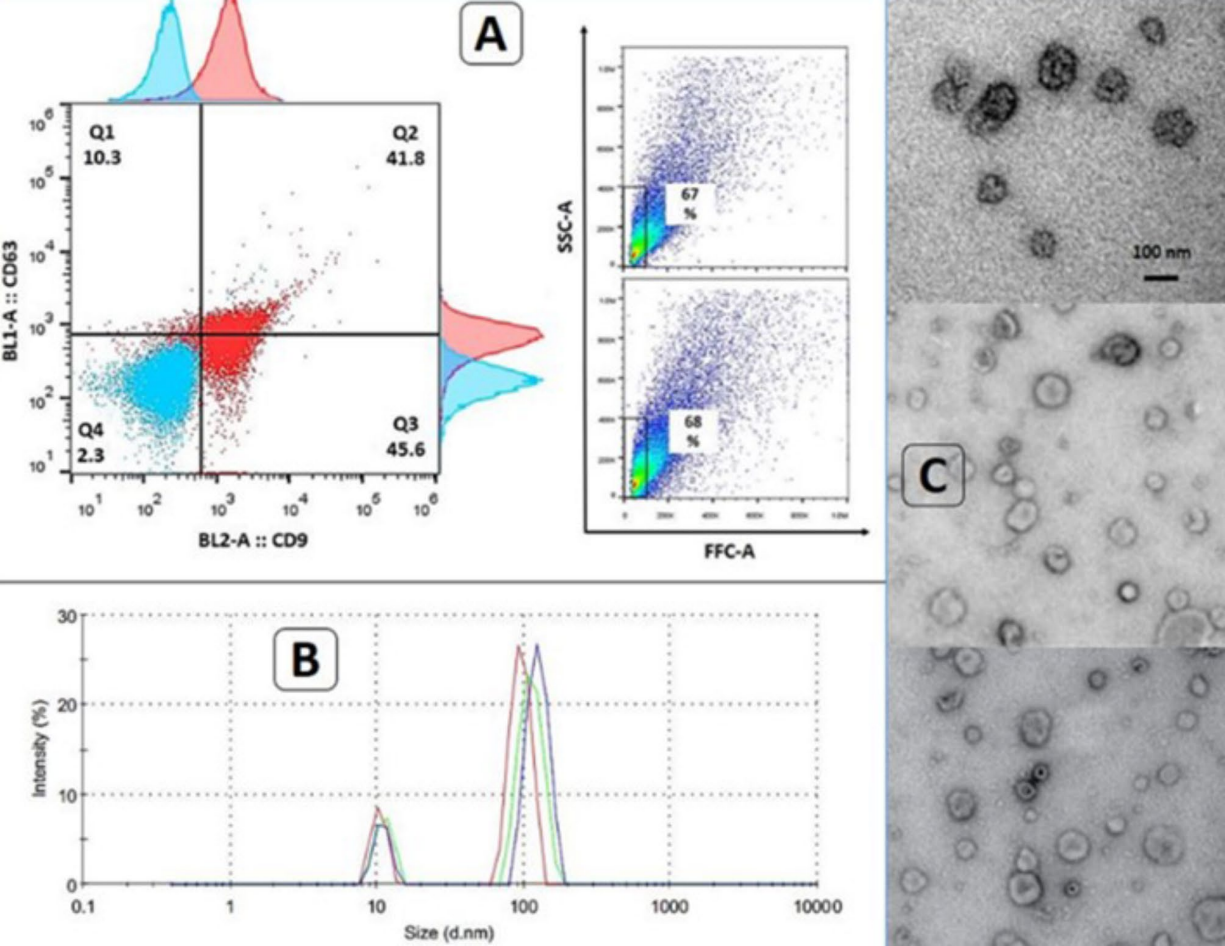
Isolated EVs confirmation. **A**: Flow cytometry for CD9 and CD63 with 87.4% and 52.1% positivity, respectively. **B**: DLS results indicated majority of EVs were ~ 100 nm. **C**: TEM images were in accordance with DLS results in term of size. **EVs**: Extracellular vesicles, **DLS**: Dynamic light scatter, **TEM**: Transmission electron microscopy

Expression of CD9 and CD63 on EVs were verified by flow cytometry (Fig. 1A). DLS analysis of isolated EVs indicated that the majority of EVs were ~ 100 nm (Fig. 1B).TEM images confirmed the morphological criteria of EVs (Fig. 1C).

### The microRNAs expression

**Fig. 2:**
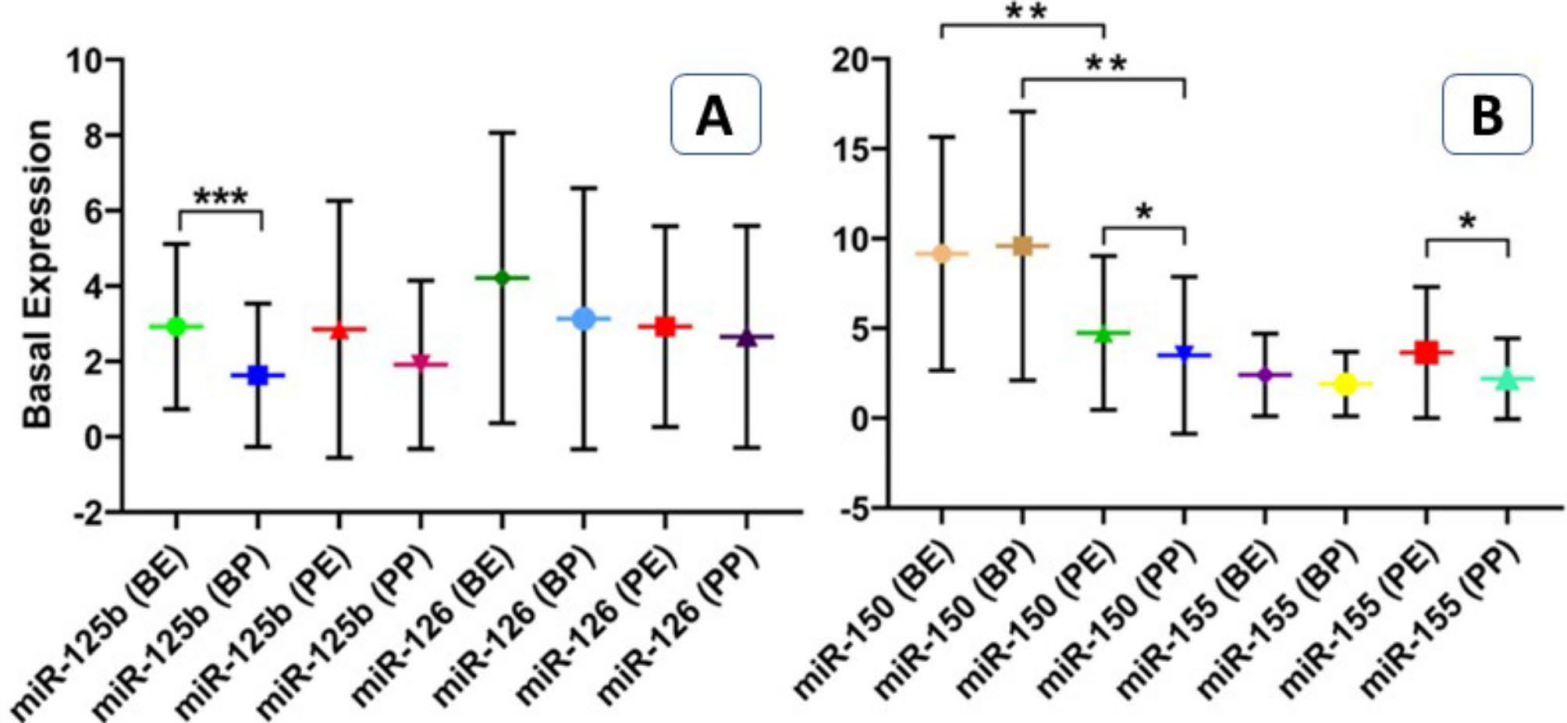
Expression of miRNAs in EVs and plasma. Expression of miR-125b, miR-126, miR-150, and miR155 in plasma and EVs, before mobilization and post conditioning were analyzed by real time polymerase chain reaction. *, ** and *** p value < 0.05, < 0.01 and < 0.001. **EVs**: Extracellular vesicles, **BE**: Before mobilization EVs, **BP**: Before mobilization plasma, **PE**: Post conditioning EVs, **PP**: Post conditioning plasma

Basal expression of four circulatory miRNAs including miR-125b, miR-126, miR-150, and miR155 in plasma and EVs, before mobilization and post conditioning were analyzed. Significantly higher expression of miR-125b was detected in EVs before mobilization in comparison with plasma (p value < 0.001) (Fig. 2A). Both miR-150 and miR-155 in EVs were significantly higher than plasma post conditioning (p value < 0.05) (Fig. 2B). There was no significant difference between before mobilization and post conditioning miRNAs, except miR-150 which was higher before than post conditioning (p value < 0.01) (Fig. 2B).

### Multi-variate and ROC analysis results

**Fig. 3:**
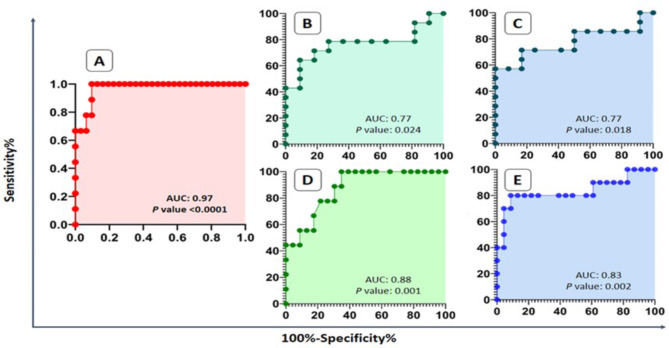
Receiver-operating characteristic (ROC) analysis. ROC curves indicated powerful predictive value of miR-125b in EVs and plasma for relapse (A), in EVs for high ESR (B) in plasma for high ESR (C), in EVs for high LDH (D), and in plasma for high LDH (E). **EVs**: Extracellular vesicles, **ESR**: Erythrocyte sedimentation rate, **LDH**: Lactate dehydrogenase

COX and Poisson regression (multi-variate) analysis were done on all variables and all outcomes that were shown in Table 3. These covariates which could be related to outcomes like relapse, LDH, and ESR, were obtained from available medical records and tests and we wanted to evaluate these. There were valuable predictive markers for relapse, high LDH (> 280 U/L), and high ESR (> 25 mm/hour) as outcome variables (Table 3). miR-125b expression level before mobilization associated with relapse, high LDH, and high ESR level. Based on the results, miR-125b before mobilization was associated with all three outcome parameters. miR-125b before mobilization in EVs can predict relapse with HR: 2.23 and p value: 0.01, and in plasma with HR: 6.7 and p value < 0.001 (Table 3). As shown in Table 3, some other variables were significantly associated with relapse, high LDH, and high ESR through multivariate analysis. We have analyzed the predictive value of miR-125b in the prediction of the three outcomes by ROC that was shown in Fig. 3A-E. miR-125b level before mobilization in both EVs and plasma was a convenient predictor for relapse, high ESR, and high LDH after AHSCT (Fig. 3A-E) (p value: 0.0001, 0.024, 0.001, 0.018, and 0.002).

**Table 3:** Predictive variable for relapse, LDH, and ESR as AHSCT outcomes by multivariate analysis. COX regression for relapse and Poisson regression for LDH and ESR

Relapse			LDH			ESR		
Variables	HR (95% CI)	*P* value	Variables	Beta	*P* value	Variables	Beta	*P* value
**miR-125b BE**	**2.23 (2.12–2.78)**	**0.01**	Intercept	6.65	< 0.0001	Intercept	2.01	0.044
**miR-125b BP**	**6.7 (6.41–7.02)**	**< 0.001**	**miR-125b BE**	**4.79**	**< 0.0001**	**miR-125b BE**	**5.65**	**< 0.0001**
Age	0.24 (0.20–1.02)	0.426	**miR-125b BP**	**8.52**	**< 0.0001**	**miR-125b BP**	**2.41**	**0.015**
Engraftment day	1.04 (0.74–1.91)	0.091	Age	0.35	0.72	Age	1.11	0.267
Diagnosis to transplantation	0.72 (0.57–1.22)	0.372	Weight	2.45	0.064	Weight	1.65	0.098
G-CSF dose for mobilization	0.8 (0.07–1.8)	0.263	Diagnosis to transplantation	1.83	0.067	Chemotherapy Courses	1.48	0.139
CD34^+^ cell count/kg	1.01 (0.04–1.43)	0.117	Mobilization Days	4.46	< 0.1	WBC count in apheresis	0.92	0.356
			G-CSF dose for mobilization	6.28	0.1	MNC count in apheresis	1.74	0.081
			Pre-apheresis CD34^+^cell count	1.78	0.074	CD34^+^ cell count/kg	2.42	0.15
			Total processed blood in Apheresis	3.64	0.12	Conditioning days	1.35	0.177
			MNC count in apheresis	4.01	0.08	Engraftment day WBC count	1.97	0.068
			**CD34** ^**+**^ **cell count/kg**	**3.02**	**0.0026**	Engraftment day PLT count	1.63	0.103
			Conditioning days	2.07	0.38	Engraftment Day	2.34	0.19
			**Engraftment day**	**4.50**	**< 0.0001**	miR-155 PE	0.69	0.489
						miR-155 BP	1.26	0.207
						miR-126 BP	1.17	0.07
						miR-126 PP	0.09	0.926

**ESR**: Erythrocyte sedimentation rate, **LDH**: Lactate dehydrogenase, **EVs**: Extracellular vesicles, **HR**: Hazard ratio, **BE**: Before mobilization EVs, **BP**: Before mobilization plasma, **PE**: Post conditioning EVs, **PP**: Post conditioning plasma, **WBC**: White blood cell, **MNC**: Mono nuclear cell, **G-CSF**: Granulocyte colony stimulating factor

### RFS and CI of outcomes

**Fig. 4:**
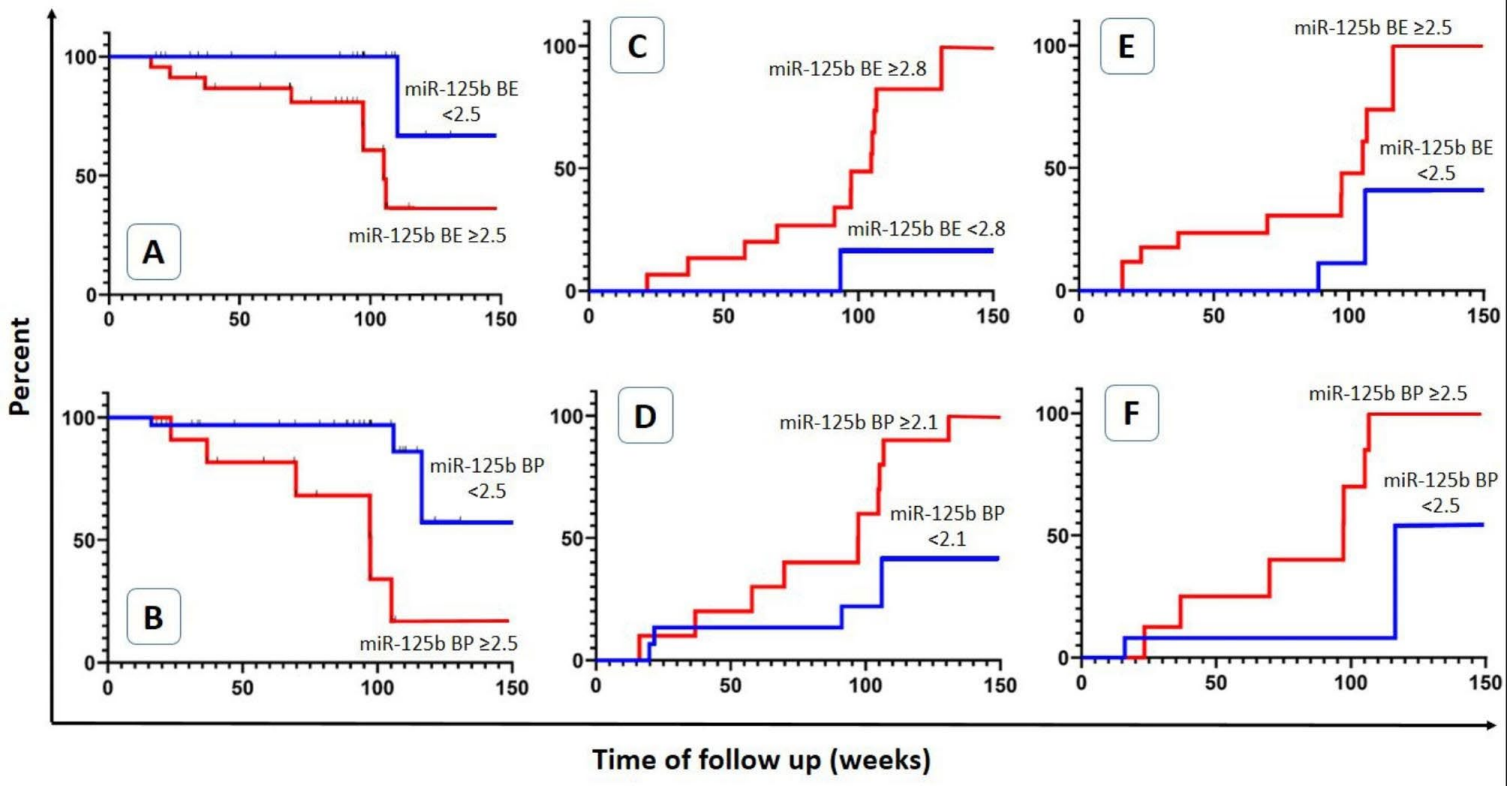
Relapse free survival (RFS) analysis and cumulative incidence by Kaplan-Meier test. Decreasing RFS is associated with increased expression of miR-125b (**A** & **B**), and high LDH (**C** & **D**) and high ESR (**E** & **F**) associated with increased expression of miR-125b (more than cut off). **BE**: Before mobilization EVs, **BP**: Before mobilization Plasma. p values **A**: 0.006, **B**: 0.0003, **C**: 0.04, **D**: 0.03, **E**: 0.04 and **F**: 0.0003

RFS and CI of high LDH (> 280 U/L), and high ESR (> 25 mm/hour) based on miR-125b expression before mobilization was evaluated by the Kaplan-Meier test (Fig. 4A-F). RFS analysis show increased incidence of relapse (decrease in RFS) after AHSCT increases in miR-125b expression ≥ 2.5 in plasma and EVs, significantly (p value: 0.006 and 0.0003) (Fig. 4A and B). Based on CI, with an increase of miR-125b expression, high LDH, and high ESR will be increased. More LDH after AHSCT was significantly associated with miR-125b expression of more than 2.1 in plasma (p value: 0.04 and 0.03) (Fig. 4C and D). Also, increasing miR-125b expression by more than 2.5 in plasma and EVs, resulted in a significantly increasing in ESR after AHSCT (p value: 0.04 and 0.0003) (Fig. 4E and F).

## Discussion

Therapeutic options for relapsed Hodgkin and non-Hodgkin lymphoma are so limited, except allogeneic HSCT with some challenges like graft versus host disease [16]. There is no standard treatment for relapsed MM after AHSCT [17]. So, the detection of predicting factors for relapse can be applicable to the prevention of it and therapy modification at AHSCT time [18]. Also, there are some strategies after AHSCT for improving the outcomes, if physicians are notified in time by some markers [18]. In our study, circulatory miRNAs and many variables were evaluated to their probable predictive potential of AHSCT and relapse-related outcomes. We found that circulatory miR-125b expression level in EVs and plasma is a strong predictor for relapse after AHSCT and also, LDH and ESR are as informative factors of risk, prognosis, and relapse of MM and lymphoma. In MM patients with upregulated miR-125b, event-free survival is shorter significantly [19]. miR-125b reduces the metastasis-associated lung adenocarcinoma transcript 1 (MALAT1) gene expression via neurogenic locus notch homolog protein 1 (NOTCH1) signaling pathway in MM cells, results in more progressiveness of MM and it can be a therapeutic target [20]. Indeed miR-125b, as an onco-miR, targets tumor suppressors including long non-coding RNA named insulin-like growth factor 1 receptor antisense imprinted non-protein coding RNA (IRAIN) and PH domain and leucine-rich repeat protein phosphatase 2 (PHLPP2) that finally elevates AKT signaling pathway and myeloma cell progression [20, 21]. However, miR-125b is a multifunctional miRNA [22] with a positive effect on dexamethasone [23] and stress adaptation induced myeloma cell death [24]. Due to the high expression of miR-125b in EVs [25], its up-regulation in serum associated with Rituximab, Cyclophosphamide, Hydroxy daunorubicin, Vincristine, and Prednisone (R-CHOP) resistance, further relapse, and diminished survival in diffuse large B cell lymphoma patients [26]. Nuclear factor kappa-light-chain-enhancer of activated B cells (NFkB) activity in B cell lymphoma increases by miR-125b that targets the tumor necrosis factor alpha-induced protein 3, thereby facilitating the cancer progression and cell proliferation [27, 28]. Totally, silencing of miR-125b is required for normal hematopoietic stem cell development toward B lymphocyte and neoplasia suppression [29]. These published studies indicate that mir-125b induces the progression and relapse of MM and lymphoma. Based on our hypothesis, circulatory miR-125b expression level might be able to the prediction of relapse after AHSCT. We introduce miR-125b as a potent predictor of relapse after AHSCT, and also a good predictor of LDH and ESR. Although high LDH is known as a bad prognosis marker in newly diagnosed MM patients [30], it is a valuable informative parameter about outcomes and survival after allogeneic HSCT for MM [31]. LDH concentration should be considered as an applicable variable for risk stratification of MM [32], as a high probability of relapse after two years of AHSCT in patients with a high level of LDH is reported [33]. In addition, high concentrations of calcium and LDH (> 240 U/L) are criteria for stage III of the international staging system with elevated relapse risk of MM [33]. In the study, we considered high LDH as more than 280 U/L. Relapse and mortality after AHSCT is predicted by increased LDH (over a period of three months) in patients with lymphoma [34, 35].Through LDH along with ESR, survival analysis and monitoring of therapy in MM and Hodgkin lymphoma will be reliable, respectively [36, 37]. Elevated ESR after treatment like radiotherapy, chemotherapy, and HSCT is associated with relapse of Hodgkin lymphoma [38, 39]. So, evaluation of LDH and ESR can be valuable for the prediction of relapse of MM and lymphoma after AHSCT. Not only the evaluation of these parameters by themselves, weeks after AHSCT but also the measurement of associated parameters like miRNAs at the time of AHSCT could be informative about relapse and its predictive markers (LDH and ESR). Indeed miR-125b is able to prediction of relapse much earlier than LDH and ESR [40]. According to the effect of miR-125b on MM and lymphoma, administration of miR-125b antagomir inhibits cell proliferation, tumor growth, and neoplasm relapse [40].

## Conclusion

As a conclusion circulatory miR-125b is associated with the risk of relapse and relapse-related parameters in MM and lymphoma patients. Monitoring of the circulatory level of miR-125b at the AHSCT time predicts the treatment response and helps to do additional strategies for the prevention of relapse.

## Data Availability

The data have included in the text and documented/reserved with the corresponding author.

## Abbreviations

AHSCT: Autologous hematopoietic stem cell transplantation
MM: Multiple myeloma
miRNAs or miR: MicroRNAs
SOX30: SRY-box transcription factor 30
NK/T: Natural killer/ T cell
PI3K/AKT/mTOR: Phosphoinositide 3-kinases/ Serine-threonine protein kinas/ Mechanistic target of rapamycin kinase
G-CSF: Granulocyte colony stimulating factor
CEAM: Lomustine, Etoposide, Cytarabine, Melphalan
BEAM: Carmustine, Etoposide, Cytarabine, Melphalan
PBS: Phosphate buffer solution
DLS: Dynamic light scatter
TEM: Transmission electron microscopy
PCR: Polymerase chain reaction
CT: Cycle threshold
CBC: Complete blood count
ESR: Erythrocyte sedimentation rate
CRP: C-reactive protein
LDH: Lactate dehydrogenase
STATA: South texas art therapy association
HR: Hazard ratio
ROC: Receiver-operating characteristic
CI: Cumulative incidence
BMI: Body mass index
WBC: White blood cell
MNC: Mono nuclear cell
CPK: Count per kilogram
PAPBS: Pre-apheresis peripheral blood sample
Ca: Calcium
Cr: Creatinine
ALP: Alkaline phosphatase
BE: Before mobilization EVs
BP: Before mobilization plasma
PE: Post conditioning EVs
PP: Post conditioning plasma
MALAT1: Metastasis associated lung adenocarcinoma transcript 1
NOTCH1: Neurogenic locus notch homolog protein 1
CHOP: Rituximab, Cyclophosphamide, Hydroxy daunorubicin, Vincristine, and Prednisone
NFkB: Nuclear factor kappa-light-chain-enhancer of activated B cells
IRAIN: Insulin like growth factor 1 receptor antisense imprinted non-protein coding RNA
PHLPP2: PH domain and leucine rich repeat protein phosphatase 2
AKT: Serine-threonine protein kinas
